# Patients’ Perspectives Regarding Digital Health Technology to Support Self-management and Improve Integrated Stroke Care: Qualitative Interview Study

**DOI:** 10.2196/42556

**Published:** 2023-04-04

**Authors:** Esmee L S Bally, Demi Cheng, Amy van Grieken, Mireia Ferri Sanz, Oscar Zanutto, Aine Carroll, Andrew Darley, Bob Roozenbeek, Diederik W J Dippel, Hein Raat

**Affiliations:** 1 Department of Public Health Erasmus MC University Medical Center Rotterdam Netherlands; 2 R&D+i Consultancy Kveloce I+D+i Valencia Spain; 3 European Project Office Department Istituto per Servizi di Ricovero e Assistenza agli Anziani Treviso Italy; 4 School of Medicine University College Dublin Dublin Ireland; 5 Academic Department National Rehabilitation University Hospital Dublin Ireland; 6 Department of Neurology Erasmus MC University Medical Center Rotterdam Netherlands

**Keywords:** stroke patients, digital health technology, self-management, co-design, user-requirements, user-centered design, qualitative research

## Abstract

**Background:**

Digital technologies such as mobile apps and robotics have the potential to involve stroke patients better in the care process and to promote self-management. However, barriers exist that constrain the adoption and acceptance of technology in clinical practice. Examples of barriers are privacy concerns, challenges regarding usability, and the perception that there is no need for health-related technology. To address these barriers, co-design can be used to enable patients to reflect on their experiences of a service and to tailor digital technologies to the needs and preferences of end users regarding content and usability.

**Objective:**

This study aims to explore the perspectives of stroke patients toward how digital health technology could support self-management regarding health and well-being, as well as integrated stroke care.

**Methods:**

A qualitative study was conducted to understand patient perspectives. Data were collected in co-design sessions during the ValueCare study. Patients from a Dutch hospital who experienced an ischemic stroke (n=36) within the past 18 months were invited to participate. Data collection took place between December 2020 and April 2021 via one-to-one telephone interviews. A short self-report questionnaire was used to collect data on sociodemographics, disease-specific information, and technology use. All interviews were audio-taped and transcribed verbatim. The interview data were analyzed using a thematic approach.

**Results:**

Patients held mixed attitudes toward digital health technologies. Some patients viewed digital technology as a convenient product or service, while others expressed no desire or need to use technology for self-management or care. Digital features suggested by stroke patients included (1) information about the causes of stroke, medication, prognosis, and follow-up care; (2) an online library with information regarding stroke-related health and care issues; (3) a personal health record by which patients can retrieve and manage their own health information; and (4) online rehabilitation support to empower patients to exercise at home. Regarding the user interface of future digital health technology, patients emphasized the need for easy-to-use and simple designs.

**Conclusions:**

Stroke patients mentioned credible health information, an online library with stroke-related health and care information, a personal health record, and online rehabilitation support as the main features to include in future digital health technologies. We recommend that developers and designers of digital health for stroke care listen to the “voice of the stroke patients” regarding both functionality and the characteristics of the interface.

**International Registered Report Identifier (IRRID):**

RR2-10.1186/s12877-022-03333-8

## Introduction

Stroke is a leading cause of death and long-term disability [1]. In 2017, there were an estimated 9.53 million prevalent stroke cases in the European Union, and this number is expected to rise to 12.11 million by 2047 [2]. Stroke patients often experience long-lasting physical and psychological consequences after stroke that can result in disruption of cognitive and emotional functioning and social relationships [3-5]. Postacute stroke care aims to support restoration of a patient’s functioning, including access to ongoing diagnostics, therapy, rehabilitation, psychological support, and self-management strategies [6].

Rehabilitation, including physical therapy, speech and language therapy, and occupational therapy, can improve mobility, communication skills, and activities of daily living in stroke patients [7,8]. The Action Plan for Stroke Europe recommends a documented plan for community rehabilitation and self-management support for all stroke patients including periodic reviews to adjust rehabilitation and other needs over time [6]. However, due to budget constraints, rehabilitation in inpatient facilities is often restricted to a few weeks, and resources are limited in long-term outpatient rehabilitation [9].

In response to these challenges, digital health technology could support existing clinical practice, as it provides opportunities to involve patients in the care and decision-making process and to promote self-management among patients [10,11]. The World Health Organization has defined digital health as “the field of knowledge and practice associated with the development and use of digital technologies to improve health” [12]. It includes a wide range of digital technologies for health such as information and communication technology, mobile wireless technology, artificial intelligence, big data, and robotics [12].

One example is telerehabilitation for stroke care that can be delivered via robotics, virtual reality, commercial gaming devices, and communication tools such as videoconferencing and telephoning [11]. It can be used to make rehabilitation training accessible for patients, especially for those living in remote areas [9,13]. A recent Cochrane review found moderate-level evidence that telerehabilitation is more effective or similarly effective as in-person rehabilitation [14].

In addition, previous studies indicate that mobile apps can support patients by, for example, acting as physical activity monitors to avoid sedentary behavior [15], providing content for stroke education [16], and sending medication reminders through text messaging [17]. The majority of commercial apps designed specifically for stroke patients or their caregivers focus on activities to help improve language and communication difficulties [18]. Furthermore, digital health technologies could support successful integrated care by facilitating good communication of information with the patient and between stroke care providers [19]. The involvement of various disciplines, institutions, and organizations in stroke care, such as hospitals, rehabilitation centers, and home care providers, requires processes of linking and coordinating services to overcome fragmentation.

Previous pilot studies on digital health interventions for stroke patients suggest that technology could be a meaningful tool for postacute stroke care [20-22]. However, there might be barriers constraining the adoption and acceptance of technology in clinical practice and by end users; these barriers include privacy concerns, challenges regarding the usability, and the perception that there is no need for health-related technology [10,23]. In this regard, co-design enables patients, their caregivers, and health care staff to reflect on their experiences with a service and to identify improvement priorities [24]. Furthermore, co-design ensures digital technologies are tailored to the needs and preferences of end users regarding content and usability [25].

As part of the ValueCare study [26], a co-design approach was undertaken to develop value-based integrated care supported by digital technologies. This study used data from co-design sessions with stroke patients. The aim of this study was to explore perspectives of stroke patients toward how digital health technology could support self-management regarding health and well-being, as well as integrated stroke care.

## Methods

### Study Design

A qualitative study design was undertaken [27]. We conducted a semistructured interview study to gain an in-depth understanding of patient perspectives [28]. Semistructured interviews were used to ensure a flexible structure of follow-up questions in exploring patients’ thoughts and experiences [29]. This study was conducted in Rotterdam, The Netherlands, as part of the larger ValueCare study [26]. The ValueCare project aims to develop and implement efficient, outcome-based, integrated health and social care for older people with multimorbidity, frailty, or mild to moderate cognitive impairment in 7 sites (Athens, Greece; Coimbra, Portugal; Cork/Kerry, Ireland; Rijeka, Croatia; Rotterdam, The Netherlands; Treviso, Italy; and Valencia, Spain). Each site is expected to adapt the general value-based methodology to their target group and local context. In order to have an in-depth, multifaceted exploration of stroke patients’ perspectives, this study solely focused on the data collected at the Rotterdam site.

Due to the COVID-19 pandemic, all interviews were conducted by telephone. Included questions were reviewed and discussed by the ValueCare consortium to fulfill the project requirements. Subsequently, interview questions were adapted to the local pilot site context. An interview guide of 5 questions (see Multimedia Appendix 1) was used for the interviews, which were iterative in nature. The interview started by asking patients to describe the onset of stroke and how they experienced received care. Subsequent questions explored their values and needs regarding postacute stroke care and how stroke care can be improved, particularly with help from digital technologies. This study focused on 1 open-ended question: How can digital solutions support you to manage your health and care? We asked participants to share their associations regarding digital solutions used in health care and what would be useful for them. Several follow-up questions regarding the characteristics of the technology and foreseen barriers to use of the technology were asked to deepen the conversation (see Multimedia Appendix 1).

### Recruitment

Patients were purposively sampled from a single-site, large, academic hospital in Rotterdam, The Netherlands. Purposive sampling was used to include patients with a variety of background characteristics in terms of sex, age, time since stroke, and severity of stroke [30]. Patients’ eligibility for the study was assessed by a physician-researcher of the Department of Neurology by screening electronic patient files. The inclusion criteria were as follows: (1) diagnosed with ischemic stroke (first ever or recurrent) within the past 18 months at the time of recruitment, (2) community-dwelling (not in long-term care) at the time of recruitment, and (3) able to provide written consent to participate in this study. We aimed to avoid excluding patients with cognitive or communication deficits, for example, by allowing support from the (informal) caregiver when communication was slow. Therefore, patients were excluded only if they did not speak Dutch or were unable to communicate sufficiently to participate. Exclusion was determined at the time of the interview.

In collaboration with the Department of Neurology, 310 patients were invited to join the study. An information package with an invitation letter, information sheet, informed consent form, and prepaid envelope was distributed by post to eligible patients. Patients were invited to share their care experiences and to provide suggestions on how to improve stroke care in general and with the support of digital technologies specifically. Interested patients who returned the signed informed consent form to the researchers were contacted to plan the interview. Recruitment continued until a diverse sample with maximum variation was achieved. We aimed for balanced participation of men and women with at least one-half of the participants aged 70 years and older and inclusion of patients with a poor health status, recurrent stroke, or severe stroke. This resulted in a sample of 42 interviews. From the interviews available (n=42), 6 interviews were excluded due to poor audio quality. Finally, 36 interviews were included in the analysis taking into consideration the depth and duration (>10 minutes) of the interview.

### Data Collection

Patients were interviewed between December 2020 and April 2021 by the first author (ELSB) and a research assistant. As part of the interview, patients were asked to complete a short questionnaire about their characteristics, including sex assigned at birth (male/female), age, living situation, time since stroke, first ever or recurrent stroke, perceived health, and technology use. Interviews lasted between 12 minutes and 38 minutes (24 minutes on average), were audio-recorded, and were transcribed verbatim resulting in 284 pages of transcribed material, of which 60 pages were about digital technologies.

### Data Analysis

Thematic analysis was conducted [27] using the software program NVivo, version 12. The process was based on the 6 phases of thematic analysis described by Braun and Clarke [27]: familiarization with data, generating initial codes, searching for themes, reviewing themes, defining and naming themes, and producing the final analyses. Thematic analysis allowed research findings to emerge from the raw data without imposing pre-existing assumptions on the setting under inquiry [27]. Two researchers (ELSB, DC) independently read the transcripts. Separately from each other, the researchers applied inductive coding with a focus on experiential claims, needs, and preferences regarding their health, social care, and digital health technology. Subsequently, the 2 researchers discussed initial codes and patterns in the data. Relevant coded data extracts were clustered into potential themes and subthemes. Themes were identified when they appeared consistently in a number of transcripts. Identified themes and subthemes were reviewed and discussed by the research team to ensure they were coherent. If necessary, recoding was performed. The analysis resulted in 3 final themes. A selection of quotes was translated into English using forward and backward translations.

### Ethical Considerations

The Medical Ethics Committee of Erasmus MC University Medical Center (Erasmus MC) in Rotterdam, The Netherlands, declared that the rules laid down in the Medical Research Involving Human Subjects Act (also known by its Dutch abbreviation WMO) do not apply to this research proposal (reference number: MEC-2021-0866). All participants provided written informed consent for participation in the study. To protect the privacy of participants, study data were de-identified (ie, pseudoanonymization). The contact details and research data of participants were coded and stored separately. Participants who completed the interview received a gift voucher of €15 (US $15.93) for their time and effort to participate in the study.

## Results

### Sample Characteristics

The final sample consisted of 36 participants (15 women and 21 men) with two-thirds of participants aged 70 years or older. Time since stroke onset was 1 year or more for 72% (26/36) of participants. Among the 36 participants, 30 (83%) had their first ever stroke. Most of the participants used the internet every day (26/36, 72%), owned a smartphone or tablet (31/36, 86%), and used apps (29/36, 81%). Participant characteristics are further described in Table 1.

**Figure 1:**
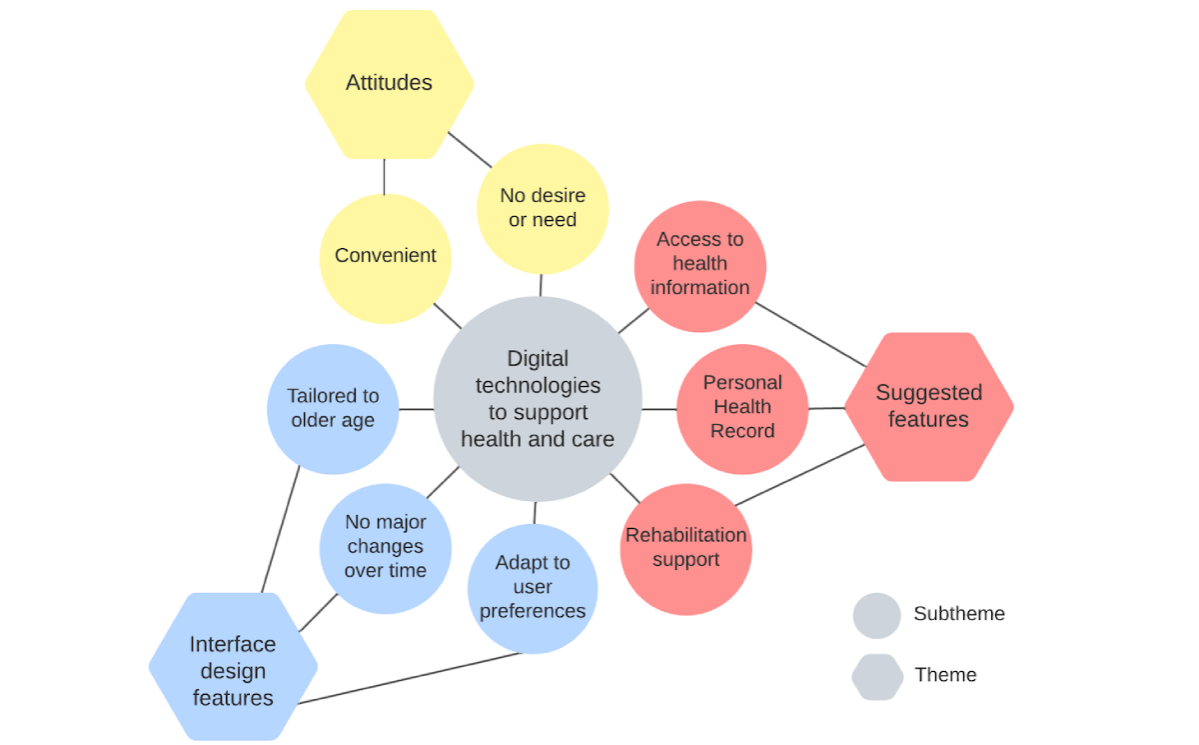
Themes and subthemes identified during data analysis.

The following themes that emerged from the interviews are described in subsequent sections: (1) attitudes toward using digital health for care, (2) suggested features of digital health technologies, and (3) suggested user interface design features of digital health technologies (see Figure 1). Barriers to the use of digital technologies have been integrated in themes 1 and 3. An overview of the identified barriers is provided in Textbox 1.

**Table 1:** Sample characteristics (n=36).

Patient	Sex	Age (years)	Time since stroke (months)	First ever stroke	Perceived health	Use of internet	Smartphone or tablet	Use of apps
P01	Male	60	12-18	Yes	Good	Every day	Smartphone	Every day
P02	Male	64	12-18	Yes	Fair	Every day	Smartphone	Multiple times a week
P03	Male	70	12-18	Yes	Good	Every day	Smartphone	Multiple times a week
P04	Male	76	12-18	Yes	Fair	Multiple times a week	Smartphone	Multiple times a week
P05	Male	83	12-18	Yes	Good	Every day	No	Never
P06	Female	71	12-18	Yes	Fair	Every day	Both	Every day
P07	Male	81	6-12	No	Good	Every day	Smartphone	Once to twice a week
P08	Male	71	6-12	Yes	—^a^	—	—	—
P09	Male	69	6-12	Yes	Good	Every day	Both	Every day
P10	Female	70	12-18	Yes	Fair	Every day	Both	Never
P11	Male	54	12-18	Yes	Good	Every day	Both	Every day
P12	Female	86	12-18	Yes	Good	Every day	Both	Every day
P13	Female	75	12-18	No	Fair	Multiple times a week	Both	Every day
P14	Male	54	6-12	Yes	Fair	Every day	Both	Every day
P15	Female	64	12-18	Yes	Fair	Every day	Both	Multiple times a week
P16	Female	70	12-18	Yes	Fair	Every day	Both	Every day
P17	Female	73	6-12	Yes	Good	Every day	Both	Every day
P18	Female	68	12-18	Yes	Good	Every day	Smartphone	Every day
P19	Female	90	12-18	Yes	—	—	—	—
P20	Female	72	12-18	Yes	Fair	Every day	Both	Every day
P21	Male	75	12-18	Yes	Fair	Multiple times a week	Tablet	Never
P22	Female	60	12-18	No	Good	Every day	Tablet	Every day
P23	Male	89	12-18	Yes	Fair	Every day	No	Once to twice a month
P24	Male	70	12-18	Yes	Good	Multiple times a week	Both	Every day
P25	Female	85	12-18	Yes	Fair	Every day	Smartphone	Once to twice a month
P26	Male	85	6-12	No	Fair	Every day	Both	Every day
P27	Female	69	12-18	Yes	Poor	Every day	Both	Every day
P28	Male	79	6-12	Yes	Fair	Multiple times a week	Both	Every day
P29	Male	73	6-12	No	Good	Every day	Both	Every day
P30	Male	82	12-18	Yes	—	—	—	—
P31	Male	75	12-18	Yes	Good	Every day	Both	Every day
P32	Female	78	12-18	No	Poor	Multiple times a week	Both	Once to twice a week
P33	Male	73	6-12	Yes	Good	Every day	Both	Every day
P34	Female	68	6-12	Yes	Fair	Once to twice a week	Smartphone	Every day
P35	Male	60	12-18	Yes	Good	Every day	Smartphone	Every day
P36	Male	66	12-18	Yes	Good	Every day	Tablet	Never

^a^Not answered.

**Barriers to the use of digital technologies.**

**Barrier**

1. No need for health-related technology
  Satisfied with received care
  Preference for physical contact with health care professional
2. Too complicated
  Missing the skills to use technology
  Challenging to get familiarized with new technologies
3. App updates
  Not able to cope with changes in a familiar interface design
4. Inflexible
  Feeling pressured and/or annoyed by push notifications
  Not wanting to depend on your phone — a phone can get lost

### Theme 1: Attitudes Toward Using Digital Health for Care

Analysis revealed mixed attitudes among patients toward using digital health to support their self-management and improve the care they receive. Most participants viewed digital health technology as a product or service, such as an online portal to manage their care, which was believed to be convenient and valued for the ability to access relevant health information:

Yes, that [patient portal] is certainly useful. I think it is quite convenient to be reminded of your doctor’s appointment the day before. I have my agenda on my iPhone to be able to receive notifications in case I forget an appointment.

P07

Other patients mentioned they experienced no desire or need to use digital health to self-manage their health and care. These participants preferred to have in-person physical contact with their health care professional rather than receiving care using technology:

I would rather have physical contact to explain what I am thinking or feeling. No, I am not in favor of technology. At least, it depends what it concerns, but with regard to my health, I prefer to have someone physically attending.

P05

You have to be able to look each other in the eyes. This allows you to see whether your complaints are taken seriously and if the physician is listening. [...] it has to be personal by talking to each other in person and not via video call.

P02

### Theme 2: Suggested Features of Digital Health Technologies

This theme consists of the features patients suggested to include in future digital solutions to support their self-management and to improve the care they receive. We identified 4 elements: (1) the need for information about the causes of stroke, medication, prognosis, and follow-up care; (2) an online library with information regarding stroke-related health and care issues; (3) a personal health record by which patients can retrieve and manage their own health information; and (4) online rehabilitation support to empower patients to exercise at home.

Some patients suggested including educational features about their condition. More specifically, they expressed the need for information about the causes of stroke, medication, prognosis, and follow-up care:

I have had this prescription from my physician. I received the medication, it had the name on it, but what does it do exactly taking such a pill?

P36

I thought: What the hell happened to me? And then they [health care professionals] are going to tell you all about it. I thought sure but I just did not know. So, in little chunks, I asked [the nurse] something every time.

P16

Patients also emphasized it was difficult to navigate the internet in their search for relevant information. An easy-to-find online library, preferably hosted by the hospital, with credible information was suggested for stroke patients:

Some sort of digital information channel which is centrally regulated by the hospital and the rehabilitation center. It should include clear information that serves the needs of stroke patients.

P22

A few patients suggested access to a personal health record to manage information about their health and care. Patients identified the potential benefits of personal health records by being able to access up-to-date information about their condition, such as medical files and prescribed medications:

I would like to see something in which you can view your medical files, but also your appointments, reminders, and a short report of the consultation you have had.

P01

Patients also discussed how digital solutions could potentially support rehabilitation at home. More specifically, patients wanted tips to increase their physical activity or support to perform exercises as part of their rehabilitation program:

I would like to have tips about exercises I can do from home. I have tried this exercising program on TV, but that is not feasible for me as my balance is not so good.

P27

In addition, participants discussed a lack of understanding regarding the exercises they are intended to perform in their home setting. A number of participants suggested that the use of visual aids (ie, pictures or videos) to explain rehabilitation exercises would help them understand and to engage with their training at home:

Usually, I recognize the exercises but sometimes I forget how to perform the exercise. For example, do I have to stand on one leg or both? [...] I like this app that shows pictures of the exercises, it also provides written text and audio explaining how to perform the exercises.

P32

### Theme 3: Suggested User Interface Design Features of Digital Health Technologies

Patients offered suggestions for the user interface of future digital solutions. Some patients expressed the need to tailor digital health technologies to older age in order to ensure technologies are acceptable to potential users:

Adapt it [the technology] to our age group. Younger people grow up with these technologies in a playful way, but I had to learn using these technologies at later age. It should not be too complicated.

P05

Participants proposed that technologies need to be designed in a way that are easy to use without consciously thinking about how to use them. This appeared to be an important factor in incorporating technology in their daily life:

There are no standards mobile apps have to comply to. For example, having always a button at the top right to log out. It depends on the developers, make it intuitive.

P01

Furthermore, participants viewed typing written text in a mobile app as difficult. Consequently, some patients preferred to use a device with a larger screen such as a tablet device or computer. Some participants suggested that it would be helpful if users could log into applications on various devices:

I have an iPhone, a small one, which means I am always pressing next to the letters with my fingers. So, to type on my phone is inconvenient. I prefer to use the tablet or computer.

P28

Another suggestion was to allow for flexibility and to ask users about their interface preferences. For example, some patients experienced push notifications of mobile apps as annoying:

I think you have to do it [being physically active] yourself. In the morning, when I go shopping, I walk my round. It is not something that has to be done, it happens automatically. Notifications won’t help much, I think. It is all on command, on time… no.

P33

Furthermore, introducing new design features in relation to technology was perceived by patients as hard to cope with. However, most patients accepted these challenges or asked a family member for assistance:

Some apps you get used to and those you like. Other apps require an update. Once the update has been completed, you do not recognize them anymore. Then I think: Oh no, I will wait for the next update because this is not working for me.

P01

## Discussion

### Principal Findings

This qualitative study provides insights from stroke patients into how digital health technology could support self-management regarding health and well-being, as well as integrated stroke care. Three themes emerged from the analysis: (1) attitudes toward using digital health for care, (2) suggested features of digital health technologies, and (3) suggested user interface design features of digital health technologies. Unlike previous studies focusing on exploring the experiences with digital technologies [10,31] or testing a prototype technology [32-34], our study adds to the literature by exploring how digital health technology should be designed in order to support patients. Stroke patients mentioned credible health information, an online library with stroke-related health and care information, a personal health record, and online rehabilitation support at home as the main features to include in future digital health technologies. Moreover, the results demonstrate that patients prefer digital technologies that are easy to use.

### Comparison With Prior Work

Consistent with previous studies, stroke patients used digital technologies, such as the computer or smartphone, to manage everyday life (eg, reminders, calendar) and to seek information [10,31]. However, the findings showed mixed attitudes of patients toward using digital health to support their self-management and to improve the care they receive. Some patients viewed digital health as a product or service that can be convenient and valuable to access relevant health information. It could be that the COVID-19 pandemic has positively altered patients’ perceptions of digital health, as during the pandemic, technology became essential to social interactions in general and patient-provider communication specifically. Other patients considered digital health as not needed and shared the concern that technology would replace physical contact with their health care professional. Previous studies indicated that experiencing the benefits of digital health technology influences its acceptance and use [35,36]. This requires that patients have knowledge on the potential benefits of digital health technology to provide assistance and support [37]. The findings of the analysis emphasized the need to communicate concrete benefits of digital health to the patient and, at the same time, reduce technology-related concerns such as challenges regarding usability [38]. The varying views also highlighted that a “one size fits all” approach is not appropriate for this patient population.

Suggested features of digital health technologies by patients included (1) the need for information about the causes of stroke, medication, prognosis, and follow-up care; (2) an online library with information regarding stroke-related health and care issues; (3) a personal health record by which patients can retrieve and manage their own health information; and (4) online rehabilitation support to empower patients to exercise at home. The findings emphasized the importance of tailoring information to patients’ needs and concerns, as described in earlier studies [39,40]. Therefore, features of digital health technologies should facilitate a personalized approach to meet individual needs. Patient portals have the potential to enhance patient engagement in managing their health by allowing access to, for example, discharge summaries, medications, lab results, and secure patient-provider communication [41]. Furthermore, patients brought forward that digital health could potentially support rehabilitation at home by using visual aids to explain and perform exercises. The use of digital health technology is proposed as a useful tool to effectively deliver rehabilitation care, including the use of brain games, virtual reality, and telerehabilitation [14,42].

Suggestions for the user interface design features of digital health technologies illustrated the need to consider older patients’ preferences in all aspects of design. Patients indicated technology should be aligned with their ability to use technology, which is consistent with other studies [37,43]. More specifically, patients emphasized the need for design elements that favor simplicity and are easy to use and intuitive. Previous studies testing the usability of digital interventions for stroke patients showed that a simple design is highly valued by patients [32,34]. In addition, some patients noted that they often felt forced to engage in new technologies by push notifications, which was perceived as inflexible. It was suggested to ask users about their interface preferences before they start using the technology. Furthermore, new design features introduced by the developers of technology were perceived by patients as hard to cope with. The large diversity in patients’ familiarity with using digital technologies has been reported in previous studies [37,44]. Understanding user characteristics of stroke patients by focusing on age-related and disease-specific ability changes, including sensory, physical, and cognitive abilities, is essential to develop user interfaces that are acceptable and engaging [45]. Providing technical support to older patients tailored to their needs can enhance their digital skills and address barriers regarding usability.

### Strengths and Limitations

This study has some limitations. First, 310 patients were sent an invitation letter for the study, and only 42 participants agreed to participate. Reasons why patients did not want to participate remain mostly unclear. Some patients indicated they were too tired to participate or did not feel a need to talk about their experiences. Although the applied method may have resulted in selection bias toward relatively healthy participants, our sample also included patients with poor self-perceived health. Second, there were no pilot interviews performed, as included questions had to be in line with project requirements. However, the semistructured nature of the interview allowed for flexibility in asking follow-up questions. In addition, we closed the interview with the following question: “Have we failed to ask any question that is important to you regarding this topic?” We recommend performing pilot interviews in future research. Finally, the study was conducted within the specific context of the Netherlands; therefore, the findings may not be transferable to other settings. The Netherlands has one of the highest smartphone use rates in Europe. In 2019, 76% of people aged 65 years to 74 years and 40% of people aged 75 years and older used social media, such as WhatsApp or Facebook [46]. To increase the generalizability of our findings, we reached variation in our sample in terms of patient characteristics (eg, sex, age, severity of stroke). We recommend replication of our findings in other countries.

A strength of this study was our exploratory approach using a rigorous qualitative methodology. This allowed patients to think freely about their needs and preferences regarding digital health without commenting on an existing prototype. However, particularly for nonfrequent users of digital technologies, it was hard to bring in their own suggestions. To address this, the interviewer asked participants to share their associations regarding digital solutions used in health care and what would be useful for them. Furthermore, our study places emphasis on the requirement to include patients early in the design process of digital interventions. This involvement is considered crucial to ensure that the intervention is meaningful to the population(s) it will serve [47].

### Future Directions

The findings of this study imply that future digital health technologies could support postacute stroke patients in managing their health and care by taking a personalized approach and adapting technologies to their abilities. In this study, input was gathered from stroke patients prior to the development of the technology product or service within the ValueCare project. Future research is needed to explore the suggested features of digital health technologies in more detail. It is recommended to use an iterative co-design approach involving relevant end users, including stroke patients, their informal caregivers, and health and social care professionals. Co-design ensures digital solutions are tailored to stroke patients’ needs and preferences regarding content and usability, as it allows for continuous feedback and interaction between designers and end users [24]. In addition, this study also identified potential barriers to using digital health technologies that can be considered during design to optimize its uptake, usability, and usefulness. Future studies with a larger variety of data could focus on subgroup analyses to explore patterns in the data in more depth. The next step within the project is to translate the concept features and user requirements that resulted from this study into improved care supported by digital health technologies for stroke patients.

### Conclusions

Variability exists in stroke patients’ perspectives toward how digital health technology could support self-management regarding health and well-being, as well as integrated stroke care. Credible health information, an online library with stroke-related health and care information, a personal health record, and online rehabilitation support at home were mentioned by patients as the main features to include in future digital solutions for stroke care. In designing digital health technologies for stroke patients, the need for simplicity should be emphasized. In addition, the findings emphasized the importance of tailoring information to patients’ needs and concerns. Our study supports that designers of digital solutions should have a holistic view and complete understandings of older stroke patients by understanding their user requirements using a co-design approach. The findings of this study provide insight in the needs and preferences of stroke patients for using digital health technologies to manage their health and care, which serve as touch points that can be explored further in co-design sessions.

## Appendix

Multimedia Appendix 1 Sample interview topic guide.

## Data Availability

The data sets generated or analyzed during the current study are available from the corresponding author on reasonable request.
