# Supplementary material for: Patients’ Perspectives Regarding Digital Health Technology to Support Self-management and Improve Integrated Stroke Care: Qualitative Interview Study
Source: J Med Internet Res. 2023 Apr 4;25:e42556. doi: 10.2196/42556 (PMC10131919; doi:10.2196/42556)
Supplement: Multimedia Appendix 1 [file jmir_v25i1e42556_app1.docx]

## Multimedia Appendix 1

### Sample interview topic guide

Introduction

- Introduction with background of the study, aims and structure of the interview.
- Check for provision of informed consent and permission for audio-taping.

Experiences and needs

- What is/was going well, considering all health and social care you currently receive/have received in relation to your stroke treatment (from stroke onset until recovery at home)?
- What is/was not going well, considering all health and social care you currently receive/have received in relation to your stroke treatment (from stroke onset until recovery at home)?
- How can stroke services across the continuum of care (hospital, rehabilitation and at home in the community) be improved?

Values and preferences

- According to you, what is important and/or valuable for stroke patients regarding treatment and care?

Digital health technology

- How can digital solutions support you to manage your health and improve the post-acute stroke care you receive?
  1. What can (future) digital health technologies do to support you?
  2. What kind of characteristics should digital health technologies have for you to use it?
  3. Do you foresee any barriers in using digital health technologies?
  4. How can we overcome barriers?

Closing

- Additional topics raised by the participant.
- Thank you statement and closing.
